# Effectiveness of Red Watermelon in Preventing Atherosclerosis Through the Role of Lipids, PCSK9, LOX-1, CD36, and ABCA1 in Wistar Rats

**DOI:** 10.3390/cimb47060433

**Published:** 2025-06-08

**Authors:** Mochamad Bahrudin, Asra Al Fauzi, Paulus Sugianto

**Affiliations:** 1 Doctoral Program of Medical Science, Faculty of Medicine, Universitas Airlangga, Surabaya 60132, East Java, Indonesia; mochamad.bahrudin-2023@fk.unair.ac.id; 2 Medical Faculty, University of Muhammadiyah Malang, Malang 65144, East Jawa, Indonesia; 3 Department of Neurosurgery, Faculty of Medicine, Universitas Airlangga, Universitas Airlangga Academic Hospital, Surabaya 60132, East Java, Indonesia; 4 Department of Neurology, Faculty of Medicine, Universitas Airlangga, Dr. Soetomo General Academic Hospital, Surabaya 60132, East Java, Indonesia; paulus.sugianto@fk.unair.ac.id

**Keywords:** red watermelon, lycopene, citrulline, LOX-1, CD36, ABCA1, PCSK9 atherosclerosis

## Abstract

Atherosclerosis is a chronic condition marked by lipid accumulation, inflammation, and endothelial dysfunction, leading to narrowed arteries and an increased risk of heart attacks and strokes. Key proteins involved in this process include PCSK9, LOX-1, ROS, CD36, and ABCA1. PCSK9 degrades LDL receptors, raising blood LDL levels, while LOX-1 and CD36 promote the uptake of oxidized LDL by macrophages, enhancing foam cell formation. ABCA1, on the other hand, facilitates cholesterol efflux to HDL, reducing atherosclerosis risk. Red watermelon (*Citrullus lanatus*), rich in lycopene, citrulline, and vitamins A, C, and E, has antioxidant and cardioprotective properties. This study aimed to explore the effects of red watermelon extract on the expression of PCSK9, LOX-1, ROS, TNFα, CD36, and ABCA1 in a Wistar rat model of atherosclerosis. In a randomized control trial, male Wistar rats were induced with a high-fat diet (margarine) and treated with red watermelon extract for four weeks. The findings showed that red watermelon extract reduced the expression of PCSK9, LOX-1, CD36, ROS, and TNFα, leading to lower LDL levels, and inhibited foam cell formation. It also increased ABCA1 expression, thus promoting cholesterol efflux and higher HDL levels. Path analysis confirmed that the anti-atherogenic effect of *C. lanatus* was primarily mediated through the PCSK9-ABCA1-FC axis. This suggests that red watermelon may serve as a natural agent for atherosclerosis prevention by regulating lipid metabolism pathways.

## 1. Introduction

Atherosclerosis is a long-term condition where fats accumulate in the artery and cause inflammation and endothelial dysfunction. Over time, it leads to the narrowing of the arteries [1,2]. Atherosclerotic diseases, particularly ischemic heart disease (IHD) and stroke, are the primary mediators of CVD burden and trends, with half of CVD deaths attributed to IHD and another quarter to ischemic stroke [3,4]. Cardiovascular diseases caused 17.8 million deaths in 2019 and will rise to 23 million by 2030, particularly among the younger age groups [3,5].

At the molecular level, the development of atherosclerosis is driven by the dysregulation of lipid metabolic proteins, including PCSK9, LOX-1, CD36, and ABCA1, which are key proteins that contribute to the atherosclerosis process and have important roles in lipid metabolism and the formation of atherosclerotic plaques. PCSK9 degrades LDL receptors, which leads to an increase in LDL levels in the blood. LOX-1 and CD36 function in the uptake of oxidized LDL (OxLDL) by macrophages, which results in a buildup of cholesterol esters in the macrophages and the formation of foam cells. In contrast, ABCA1 regulates cholesterol efflux from the macrophages to HDL particles, which clear cholesterol from the macrophages and reduce the risk of atherosclerosis [6,7].

Red watermelon contains a variety of bioactive compounds, which have antioxidant, anti-inflammatory, and cardioprotective effects, as well as modulating lipid metabolism, namely lycopene, citrulline, and vitamins A, C, and E [8,9]. Studies have shown that lycopene can decrease the expression of PCSK9 in the liver [10,11], while citrulline contributes to increased nitric oxide (NO) synthesis, which improves endothelial function and reduces the risk of atherosclerosis [12,13]. Although there is evidence on lycopene and citrulline, the combined effects of red watermelon extract on the main atherogenic proteins (PCSK9, LOX-1, CD36) and atheroprotective pathways (ABCA1) have not yet been explored in vivo. We hypothesized that red watermelon extract would deregulate PCSK9, LOX-1, and CD36 while increasing ABCA1, thereby attenuating the development of atherosclerosis in rats on a high-fat diet

## 2. Materials and Methods

The design of this study was a Randomized Controlled Trial (RCT) with the post-intervention assessment of male Wistar rats (*Rattus norvegicus*) induced with a high-fat diet through margarine supplementation and a red watermelon extract intervention for four weeks [14]. Adult male Wistar rats (age, 8–12 weeks; weight, 180–220 g) were used. Animals were housed under standard laboratory conditions (12 h light/dark cycle; temperature, 22 ± 2 °C; humidity, 50–60%) and fed ad libitum with COMFEED AD II standard diet. The margarine used contained 36% saturated fat and <1% trans-fat as per the label specification. The high-fat diet (HFD) was provided ad libitum. Feed intake was monitored daily and averaged 15 g/rat/day. Wistar mice were chosen for their well-characterized response to a high-fat diet and translational relevance to human lipid metabolism. The control group (K−) received a standard diet (COMFEED AD II) without the intervention, the atherosclerosis group (K+) was fed a high-fat diet (standard diet and margarine) to induce atherosclerosis, the atherosclerosis and watermelon extract group (P1) received a high-fat diet and the red watermelon extract intervention at a dose of 500 mg/day per rat, the atherosclerosis and watermelon extract group (P2) received a high-fat diet and the red watermelon extract intervention at a dose of 750 mg/day per rat, and the atherosclerosis + watermelon extract group (P3) was given a high-fat diet and the red watermelon extract intervention at a dose of 1000 mg/day per rat [15].

This study received ethical clearance from the Research Ethics Committee of the Faculty of Medicine, University of Muhammadiyah Malang (Approval No. E.5.a/181/KEPK-UMM/VI/2024) and was conducted according to laboratory animal welfare guidelines.

### 2.1. Watermelon Extract Preparation and LC-MS/MS Examination

#### 2.1.1. Watermelon Extract Preparation

The setabindo-1 watermelon variety was sourced from PT. Bisi Internasional Tbk, Karangploso, Malang Regency, East Java, Indonesia, and it complied with the Indonesian Minister of Agriculture Decree No. 1055/KPTA/TP.240/12/97. The red flesh of the watermelon was separated from the rind, chopped into smaller pieces, and placed in a tube. Extracts were stored at −20 °C in amber vials to protect them from light until analysis. The extract was then macerated in a 96% n-hexane, acetone, and ethanol mixture at a 2:1:1 ratio (F/S 100 mL:200 mL) for 48 h. The extract was filtered and concentrated using a rotary evaporator to obtain a thick extract.

#### 2.1.2. LC-MS/MS-Based Compound Identification

Qualitative identification of the major phytochemical constituents in *Citrullus lanatus* (red watermelon) extract was performed using liquid chromatography–tandem mass spectrometry (LC-MS/MS) in positive ion mode. The analysis employed a triple quadrupole (QqQ) mass spectrometer (TSQ Fortis, Thermo Scientific, Waltham, MA, USA) coupled with an ultra-high-performance liquid chromatography (UHPLC) system (Vanquish, Thermo Scientific). Given the inherent limitation of QqQ instruments to unit mass resolution, we adopted a targeted Selected Reaction Monitoring (SRM) approach to enhance analytical specificity. This mode enables precise detection of pre-defined precursor-to-product ion transitions, providing high selectivity and sensitivity even in complex matrices, despite the absence of high-resolution mass capability.

Chromatographic separation was carried out using a Hypersil GOLD™ C18 column (100 × 2.1 mm, 1.9 µm) maintained at 40 °C. The mobile phase consisted of 0.1% formic acid in water (A) and acetonitrile (B), delivered in a gradient mode at a flow rate of 200 µL/min. The injection volume was 2 µL, and samples were maintained at 5 °C throughout the analysis.

Compound identification was performed qualitatively, without the use of external calibration standards. Detection was based on spectral pattern matching between the observed MS/MS fragmentation profiles and those archived in the NIST Mass Spectral Library, a widely validated reference database. Identification was accepted when precursor/product ion transitions matched the library entries and characteristic fragment ions were observed with consistent retention times. This approach, although qualitative, allows the confident identification of known compounds using validated SRM transitions instead of authentic reference standards.

### 2.2. Atherosclerosis Induction

To induce atherosclerosis, rats in the K+, P1, P2, and P3 groups were fed a high-fat diet consisting of COMFEED AD II mixed with margarine in a 9:1 ratio for four weeks. Each rat was estimated to consume 15 g of feed per day. Since each cage housed six rats, the total daily feed required was 90 g (the diet consisted of 10 g margarine per 90 g feed (10% margarine by weight, not 11.1% as previously implied). The ‘9:1’ refers to the feed-to-margarine ratio), including 10 g of margarine. Previous studies reported that daily intake of 1.7 g (or 1.7 mL) of margarine per rat for 4 weeks can induce atherosclerotic changes [14]. Baseline lipid profiles were assessed before induction to ensure normal levels.

### 2.3. Histopathology and Immunohistochemistry Examination

After four weeks, the rats were euthanized using anesthesia followed by exsanguination. Carotid arteries were collected and fixed in 10% formalin. Tissue samples were fixed in 10% neutral-buffered formalin for 24–48 h at room temperature to ensure adequate fixation and preservation of tissue morphology for both histological and immunohistochemical analyses. Hematoxylin and eosin (H&E) staining was performed to assess foam cell formation and tunica intima thickness. The carotid artery was selected due to its susceptibility to atherosclerotic plaque formation under disturbed flow conditions, ease of surgical access, and consistency in anatomical landmarks compared with the aorta. Immunohistochemical analysis was conducted to evaluate the expression of PCSK9 using the Abcam LS-C820134-20 antibody, LOX-1 expression using the Thermo Fisher MA5-23895 antibody, CD36 expression using the Abcam EPR-225509-40 antibody, TNF-α expression by using the Abcan-EPR 19147 antibody, and ABCA1 expression using the LS Bio LS-C543262 antibody. Primary antibodies were diluted to 1:100 and incubated overnight at 4 °C. Positive and negative controls (tissues lacking target protein expression) were included. Foam cell formation, tunica intima thickness, and protein expression levels were analyzed using a Nikon Eclipse Ei microscope with an Optilab Camera (Optilab, Miconos, Indonesia) connected to a computer at 400× magnification. Five sections per rat and five fields per section were analyzed blindly by two independent observers using ImageJ software version 1.53t (National Institutes of Health, Bethesda, MD, USA).

### 2.4. Lipid Profile Analysis

Serum lipid concentrations were measured enzymatically using commercial assay kits and read using a spectrophotometer (ICHEM-I UBIO 535) at specific wavelengths (cholesterol: 500 nm; triglycerides: 520 nm; HDL/LDL: 546 nm). Commercial assay kits for lipid profile (cholesterol, triglycerides, HDL, LDL) were obtained from ICHEM-i UBIO 535 biochemistry analyzer (i-SENS, Seoul, Republic of Korea), catalog numbers CHOL-100, TRIG-100, HDL-100, and LDL-100 [16].

### 2.5. ROS Inspection

Oxidative stress was assessed via a serological assay using the Rat ROS1/ROS ELISA Kit (Sandwich ELISA) (LS Bio, Newark, CA, USA, Catalog No. LS-F9759).

### 2.6. Statistical Analysis

Data were presented as the mean ± standard deviation (SD). Data were analyzed using one-way ANOVA and post hoc LSD tests for focused pairwise comparisons post-ANOVA. While Tukey’s HSD controls family-wise error, LSD may increase the risk of Type I error. The justification for LSD is its higher power in small-group comparisons. Statistical analysis was performed using IBM Statistical Package for the Social Sciences (SPSS) Type 27 for Windows (IBM Corp., Armonk, NY, USA). A linear regression test was used to assess the relationships among the dependent, mediating, and independent variables. Path analysis was performed to evaluate the relationships of PCSK9, LOX-1, CD36, and ABCA1 with foam cell formation in Wistar rats treated with watermelon extract. Statistical significance was set at *p* < 0.05. Path analysis was performed using standardized regression coefficients. Goodness-of-fit indices included RMSEA = 0.045, CFI = 0.97, and TLI = 0.95, indicating a good model fit.

## 3. Results

This study was conducted to explore how red watermelon (*Citrullus lanatus*) consumption modulates the lipid profile, and the expression of PCSK9, LOX-1, CD36, ROS, TNFα, and ABCA1 so that it can inhibit the development of atherosclerosis. The study parameters were assessed in Wistar rats (Rattus norvegicus) induced with a high-fat diet using margarine.

**Figure 1:**
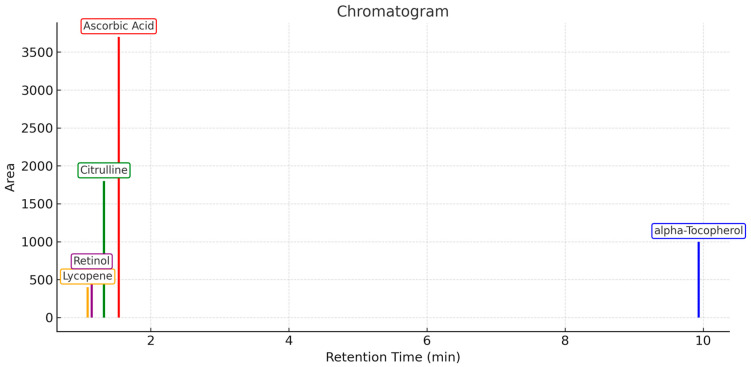
Chromatogram of *Citrullus lanatus* extract showing qualitatively identified bioactive compounds: Lycopene, Retinol, Citrulline, Ascorbic Acid, and alpha-Tocopherol. Identification was conducted using liquid chromatography-tandem mass spectrometry (LC-MS/MS) in positive ion mode with a triple quadrupole mass spectrometer (TSQ Fortis, Thermo Scientific) coupled to an ultra-high-performance liquid chromatography (UHPLC) system (Vanquish, Thermo Scientific). Chromatographic separation was achieved using a Hypersil GOLD™ C18 column (100 × 2.1 mm, 1.9 µm) at 40 °C. The mobile phase was composed of 0.1% formic acid in water (denoted as solvent A) and acetonitrile (solvent B), delivered in gradient mode at a flow rate of 200 µL/min. The injection volume was 2 µL, and the autosampler temperature was maintained at 5 °C. A targeted Selected Reaction Monitoring (SRM) approach was used to enable sensitive and specific detection of pre-defined precursor/product ion transitions. Compounds were qualitatively identified based on MS/MS spectral pattern matching with the NIST Mass Spectral Library, confirmed by characteristic fragment ions and consistent retention times. Peaks are annotated with compound names only; retention times are described in the accompanying text. The MS/MS fragmentation spectra for the identified compounds are provided as Supplementary Figure S1a–e.

The LC-MS/MS chromatogram shows the separation and detection of major bioactive compounds identified in the *Citrullus lanatus* (watermelon) extract. Peaks corresponding to ascorbic acid, citrulline, retinol (vitamin A), and lycopene were detected in the early retention time window (approximately 1.0−1.5 min), indicating their relatively high polarity and low molecular weight. In contrast, α-tocopherol appeared as a distinct and prominent peak at a higher retention time (−9.9 min), reflecting its lower polarity and higher molecular weight compared with other compounds. The intensity of each peak reflects the relative abundance of the compound in the extract, with α-tocopherol showing the most prominent peak, indicating its dominant presence in the sample. This chromatographic profile confirms the successful identification of key antioxidant and bioactive compounds in *Citrullus lanatus*, supporting its potential role in health benefits such as antioxidant activity and anti-atherogenic properties (Figure 1) [9].

Table 1 presents the effects of *Citrullus lanatus* (red watermelon fruit extract) on several atherosclerosis-related biomarkers and lipid profile parameters in a rat model. The data are expressed as the mean ± standard deviation (SD), and statistical analysis was conducted using one-way ANOVA followed by the LSD post hoc test. The expression level of PCSK9 was significantly elevated in the atherosclerosis control group (K+) compared with the negative control (K−), while the administration of *C. lanatus* extract, particularly at the highest dose (P3), significantly reduced PCSK9 levels (*p* < 0.001). Similar trends were observed for LOX-1 and CD36, where the P3 treatment led to a marked reduction in expression compared with the atherosclerosis control group (K+) and the other groups, indicating an inhibition of oxidized LDL uptake and foam cell formation.

In contrast, ABCA1 expression, which plays a protective role by facilitating cholesterol efflux to HDL, was significantly increased in the P3 group (*p* < 0.001), suggesting that *C. lanatus* supports reverse cholesterol transport. The foam cell count and tunica intima thickness, both indicators of atherogenic progression, were significantly reduced in the P3 group compared with the atherosclerosis control group (K+), indicating a protective vascular effect. Regarding the lipid profile, the P3 group exhibited significantly lower total cholesterol, triglycerides, and LDL levels and a significant increase in HDL levels compared with the atherosclerosis control group (K+) group. These findings are in line with the extract’s potential to modulate lipid metabolism and reduce atherogenic risk.

Markers of oxidative stress and inflammation, namely ROS and TNF-α, were also significantly decreased in the P3 group compared with the atherosclerosis control group (K+), further supporting the antioxidant and anti-inflammatory properties of red watermelon extract.

Overall, the post hoc analysis confirms that the highest dose of *C. lanatus* extract (P3 = 1000 mg/day/rat) consistently produced statistically significant improvements across all measured parameters when compared with the atherogenic group. These results underline the extract’s potential anti-atherosclerotic effects via modulation of key molecular pathways involved in lipid handling, oxidative stress, and inflammation.

**Figure 2:**
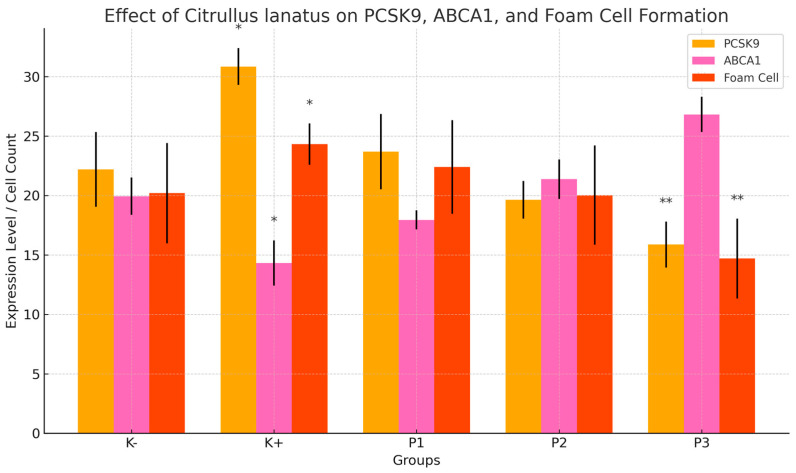
Effect of *Citrullus lanatus* (red watermelon) extracts on PCSK9, ABCA1, and foam cell formation in the carotid arteries of rats. The bar graph shows the expression levels of PCSK9 and ABCA1 (assessed by immunohistochemistry) and the number of foam cells (assessed by histopathological analysis) across different groups: normal control (K−), atherogenic control (K+), and treatment groups (P1: 500 mg/day/rat; P2: 750 mg/day/rat; P3: 1000 mg/day/rat). Data are presented as the mean ± standard deviation (SD). Error bars represent the SD. Statistical analysis was performed using one-way ANOVA followed by an LSD post hoc test. Asterisks indicate significant differences compared with the atherogenic group (K+): * *p* < 0.05; ** *p* < 0.01. The atherogenic group (K+) exhibited significantly higher PCSK9 expression and foam cell formation, but lower ABCA1 expression compared with the normal group. Treatment with *Citrullus lanatus* extract, especially at the highest dose (P3), significantly reduced PCSK9 expression and foam cell formation while increasing ABCA1 expression, indicating improved lipid metabolism and a potential anti-atherosclerotic effect.

Figure 2 illustrates the effect of red watermelon (*Citrullus lanatus*) extract on the expression levels of PCSK9 and ABCA1 and foam cell count across different experimental groups. The atherosclerosis control group (K+) exhibited significantly elevated PCSK9 and foam cell formation, alongside suppressed ABCA1 expression, indicating enhanced atherosclerotic progression. Conversely, administration of *Citrullus lanatus* extract, particularly at the highest dose (P3 = 1000 mg/day/rat), resulted in a substantial reduction in PCSK9 and foam cells, and a notable increase in ABCA1 levels. These findings suggest that the extract exerts a protective cardiovascular effect by modulating lipid metabolism and preventing foam cell formation.

### 3.1. Histopathology and Immunohistochemistry of Carotid Arteries

**Figure 3:**
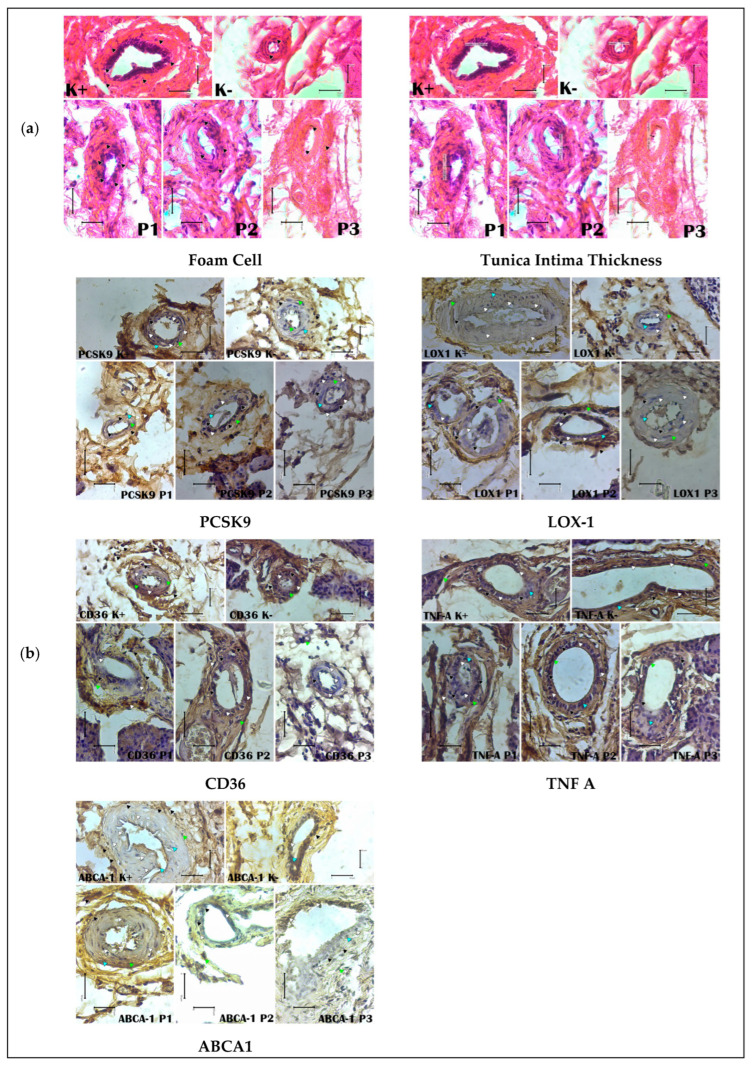
Histopathology and immunohistochemistry of carotid arteries. (**a**) Representative histological images of foam cell formation and tunica intima thickness in carotid artery sections stained with hematoxylin and eosin (H&E). Foam cells (black arrows) are indicated, and tunica intima thickness is shown in micrometers. Images were captured at 400× magnification, with scale bars representing 50 µm. Hematoxylin and eosin (H&E) staining of the carotid arteries shows prominent foam cell accumulation (black arrows) and tunica intima thickening in the atherosclerosis control group (K+), confirming the development of atherosclerotic lesions. In contrast, the treatment groups (P1, P2, P3) demonstrate a gradual reduction in foam cell formation and a thinner tunica intima compared with the atherosclerosis control group (K+), consistent with the ANOVA results showing significant differences among groups (*p* < 0.05). The post hoc LSD test revealed that P3 (1000 mg/day/rat *Citrullus lanatus* extract) significantly reduced foam cell numbers and intima thickness compared with all other groups, suggesting a dose-dependent protective effect. (**b**) Immunohistochemical staining of PCSK9, LOX-1, CD36, TNF-α, and ABCA1 in carotid artery sections. Positive cells are indicated by black arrows, negative cells by white arrows, strong immunoreaction by blue arrows, and weak immunoreaction by lime green arrows. Sections were analyzed under a light microscope at 400× magnification, with scale bars of 50 µm. Immunohistochemical analysis of the carotid arteries demonstrates the differential expression of PCSK9, LOX-1, CD36, TNF-α, and ABCA1 among groups. The atherosclerosis control group (K+) exhibited strong positive staining for pro-atherogenic markers (PCSK9, LOX-1, CD36, and TNF-α), as indicated by brown deposits (black arrows), while ABCA1, an anti-atherogenic marker, showed weaker staining. Treatment with *Citrullus lanatus* extract (P1, P2, P3) reduced the expression of PCSK9, LOX-1, CD36, and TNF-α in a dose-dependent manner, with P3 showing the lowest expression levels (confirmed by ANOVA and LSD tests, *p* < 0.05). Conversely, ABCA1 expression increased progressively across treatment groups, with P3 showing the strongest immunoreactivity, indicating enhanced cholesterol efflux capacity. These findings corroborate the biochemical results, suggesting that *Citrullus lanatus* mitigates atherosclerosis by downregulating pro-atherogenic proteins and upregulating ABCA1.

Figure 3 displays the histopathological and immunohistochemical analysis of the carotid arteries in various experimental groups, including the normal control (K−), atherogenic control (K+), and *Citrullus lanatus*-treated groups (P1, P2, P3).

Panel (a) shows representative images of foam cell formation and tunica intima thickness assessed via hematoxylin–eosin (H&E) staining. Increased foam cell formation and tunica intima thickness were observed in the atherogenic group (K+) compared with the normal control, while treatment with *Citrullus lanatus*, particularly at higher doses (P2 and P3), reduced these pathological changes.

Panel (b) displays the immunohistochemical staining of PCSK9, LOX-1, CD36, TNF-α, and ABCA1 expression. Positive staining for PCSK9, LOX-1, CD36, and TNF-α was prominent in the atherogenic group, indicating upregulation of lipid uptake and inflammatory markers. In contrast, the expression of ABCA1, a key regulator of cholesterol efflux, was markedly increased in the *Citrullus lanatus*-treated groups, suggesting improved reverse cholesterol transport.

Quantification of staining intensity was performed using image analysis software, and statistical significance was determined using one-way ANOVA with the LSD post hoc test. Asterisks indicate statistical differences compared with the atherogenic group (*p* < 0.05).

### 3.2. Path Analysis

Path analysis demonstrates the interrelationships of *Citrullus lanatus* (CL), PCSK9, LOX-1, CD36, ABCA1, and foam cell (FC) formation in atherosclerosis. The analysis reveals that CL exerts both direct and indirect effects on FC formation. Green arrows represent statistically significant pathways (*p* < 0.05), while red arrows indicate non-significant pathways (*p* > 0.05). Beta coefficients (B) and *p*-values (*p*) are displayed along each path, and arrow thickness corresponds to the magnitude of the relationship. The results indicate that CL has a significant direct inhibitory effect on FC formation (B = −0.632, *p* = 0.004) and an additional indirect effect through PCSK9 and ABCA1. Specifically, CL downregulates PCSK9 expression (B = −0.743, *p* = 0.000), which, in turn, suppresses ABCA1 (B = −0.822, *p* = 0.000), and ABCA1 exerts a protective role by reducing FC formation (B = −0.475, *p* = 0.049).

Conversely, the indirect effects of CL mediated through LOX-1 (B = 0.019, *p* = 0.920) and CD36 (B = −0.266, *p* = 0.133) were not statistically significant, suggesting that the PCSK9-ABCA1 axis plays a dominant role in modulating foam cell formation. Notably, LOX-1 and CD36, although traditionally recognized as pro-atherogenic markers, did not significantly contribute to FC modulation in this model.

The model demonstrates a high explanatory power with an R^2^ of 0.717, indicating that 71.7% of the variance in FC formation is accounted for by CL, PCSK9, ABCA1, LOX-1, and CD36, while the remaining 28.3% may be attributed to other factors not investigated in this study.

These findings highlight the potential anti-atherogenic effects of *Citrullus lanatus*, primarily through modulation of PCSK9 and ABCA1, and suggest a minor role for LOX-1 and CD36 in this pathway.

This study demonstrates the anti-atherosclerotic potential of *Citrullus lanatus* (red watermelon extract) in a rat model. Administration of *C. lanatus*, particularly at the highest dose (P3 = 1000 mg/day/rat), significantly improved lipid profiles (decreasing total cholesterol, triglycerides, LDL, and increasing HDL), reduced oxidative stress (ROS) and inflammation (TNF-α), and attenuated histopathological markers of atherosclerosis, including foam cell formation and tunica intima thickness. Molecular analyses revealed that *C. lanatus* downregulated PCSK9, LOX-1, CD36, and TNF-α expression, while upregulating ABCA1, suggesting enhanced cholesterol efflux and reduced foam cell formation.

Path analysis further confirmed that the anti-atherogenic effect of *C. lanatus* was primarily mediated through the PCSK9-ABCA1-FC axis, with a significant direct inhibitory effect on foam cell formation (B = −0.632, *p* = 0.004). The contributions of LOX-1 and CD36 were not statistically significant, indicating a minor role in this model. Overall, these findings suggest that *C. lanatus* exerts protective cardiovascular effects by modulating key molecular pathways involved in lipid metabolism, oxidative stress, and inflammation.

## 4. Discussion

This study aimed to qualitatively identify key bioactive compounds in red watermelon extract using LC-MS/MS operated in SRM mode on a triple quadrupole platform. While QqQ systems do not offer high-resolution mass accuracy, their ability to selectively monitor specific ion transitions via SRM provides robust compound-level identification when authentic standards are unavailable. This technique is particularly suited for targeted metabolite screening in complex biological or plant matrices.

The identification strategy relied on matching both the precursor and product ions, along with consistent retention times and fragmentation patterns, to entries from the NIST MS/MS library. This method offers high specificity despite the QqQ system’s limited resolving power and is commonly used in early-stage phytochemical profiling studies, where the objective is to confirm compound presence rather than quantify absolute concentrations.

Compounds including lycopene, citrulline, retinol, ascorbic acid, and α-tocopherol were all detected on the basis of diagnostic transitions and characteristic fragment ions. Their presence supports the biological observations obtained from the in vivo study, particularly regarding the modulation of lipid metabolism and oxidative stress pathways.

While this study did not include quantitative calibration, the qualitative detection of structurally confirmed compounds provides a sufficient basis to associate the extract’s observed biological activity with its constituent phytochemicals. Future work will incorporate absolute quantification using external standards, enabling a dose–response evaluation and mechanistic correlation between compound concentrations and biological outcomes.

The results of the qualitative LC-MS/MS examination of red watermelon extract confirmed the presence of lycopene, citrulline, vitamin A, vitamin C, and vitamin E. These compounds exhibit significant antioxidant and anti-inflammatory effects, which are relevant in the modulation of atherosclerosis-related pathways, particularly PCSK9 expression and cholesterol metabolism (Figure 1). Watermelon extract has the potential to affect PCSK9 due to its lycopene content. Lycopene is a well-known antioxidant compound that has been shown to reduce oxidative stress, a key trigger in the activation of inflammatory pathways including NF-κB. Oxidative stress can upregulate PCSK9 expression, which then promotes the degradation of LDL receptors (LDLR), elevates plasma LDL cholesterol levels, and enhances cardiovascular risk. Studies have indicated that lycopene inhibits the NF-κB signaling cascade, possibly by suppressing IκBα phosphorylation and preventing nuclear translocation of NF-κB. This inhibition may lead to reduced PCSK9 expression, improved LDL clearance, and an overall better lipid profile [3,6,15]. Additionally, lycopene supports healthier lipid metabolism, which indirectly modulates PCSK9 activity [3,17,18].

Citrulline, another bioactive compound in red watermelon, increases the production of nitric oxide (NO), which supports healthy blood vessel function and reduces systemic inflammation [18]. NO improves endothelial function by promoting vasodilation, reducing vascular inflammation, and inhibiting leukocyte adhesion. The anti-inflammatory effects of NO are also mediated via inhibition of NF-κB activity. Thus, citrulline indirectly contributes to the suppression of PCSK9 through improved vascular health and reduced inflammation [12,13,19].

The antioxidant effects of lycopene and the benefits of citrulline contribute to the modulation of PCSK9 activity. Thus, the consumption of red watermelon has the potential to support better cholesterol metabolism and lower the risk of diseases associated with high cholesterol levels, such as atherosclerosis. Inflammation and endothelial dysfunction often trigger lipid metabolism disorders, which increase the expression of PCSK9 as an adaptive response. Citrulline, through increased NO, can reduce inflammation and improve lipid function, so that PCSK9 expression is more controlled [18,20,21].

Vitamin C in watermelon also acts as a potent antioxidant, mitigating the generation of reactive oxygen species (ROS) and subsequently reducing oxidative stress. This effect plays a crucial role in preventing the activation of inflammatory pathways such as NF-κB. By reducing ROS and inhibiting NF-κB activation, vitamin C can lower PCSK9 expression and help maintain a normal lipid metabolism. Chronic inflammation is often associated with an increase in PCSK9 expression. The compounds in watermelon, including vitamin C and carotenoids, have anti-inflammatory properties that suppress PCSK9 expression. Chronic inflammation is often driven by oxidative stress, impaired lipid metabolism, obesity, and insulin resistance. Activation of the inflammatory pathways, such as the NF-κB pathway, triggers the production of inflammatory molecules, including PCSK9. Excess PCSK9 contributes to the degradation of LDL receptors (LDLR) in the liver, so LDL cholesterol levels in the blood increase and can worsen the risk of cardiovascular disease [3,17]. Vitamin C is an antioxidant that aids in reducing free radicals and oxidative stress, which is one of the main triggers of chronic inflammation. By lowering oxidation levels in the body, vitamin C inhibits the activation of inflammatory pathways such as NF-κB and potentially decreases the expression of PCSK9 [7,22].

The lycopene, citrulline, and vitamin C detected in red watermelon are known to have an important role in modulating the NF-κB signaling pathway, which is a key regulator in the inflammatory response. Lycopene has been reported to inhibit NF-κB activation through a mechanism of oxidative stress reduction and inhibition of IκBα phosphorylation, thereby preventing the translocation of NF-κB to the cell nucleus. Meanwhile, citrulline, as a precursor of L-arginine, plays a role in increasing the production of NO (nitric oxide), which can suppress NF-κB activation indirectly through antioxidant and vasodilation mechanisms. On the other hand, vitamin C acts as a powerful antioxidant capable of reducing the formation of ROS (reactive oxygen species), which is the main trigger of NF-κB activation. The synergy of these three compounds has the potential to decrease the expression of pro-inflammatory genes such as TNF-α and IL-6, thus contributing to the anti-inflammatory effects and anti-atherogenic potential of red watermelon.

Our experimental findings support these mechanisms. Compared with the positive control group, the group administered the highest dose of red watermelon extract (P3) exhibited significantly lower levels of PCSK9, ROS, TNF-α, and LDL. These results demonstrate the extract’s ability to suppress inflammation and improve cholesterol profiles via the combined action of lycopene, citrulline, and vitamin C (Table 1 and Figure 2 and Figure 3).

PCSK9 targets LDLR in the hepatocytes and affects macrophage function. PCSK9 can increase the expression of LOX-1, SR-A, and CD36 receptors, three key receptors involved in oxidized cholesterol uptake. These receptors play a major role in the accumulation of lipids within the macrophages. OxLDL is an important component in the formation of foam cells [6,7,23]. Increased expression of PCSK9 accelerates lipid absorption and cholesterol accumulation, which then leads to the transformation of macrophages into foam cells [7,24]. PCSK9 inhibitors will suppress SR-A, CD36, and LOX-1. Lower expression of PCSK9 could prevent foam cell formation and cholesterol accumulation in the macrophages, and ultimately reduce the risk of atherosclerosis [6,25].

**Figure 4:**
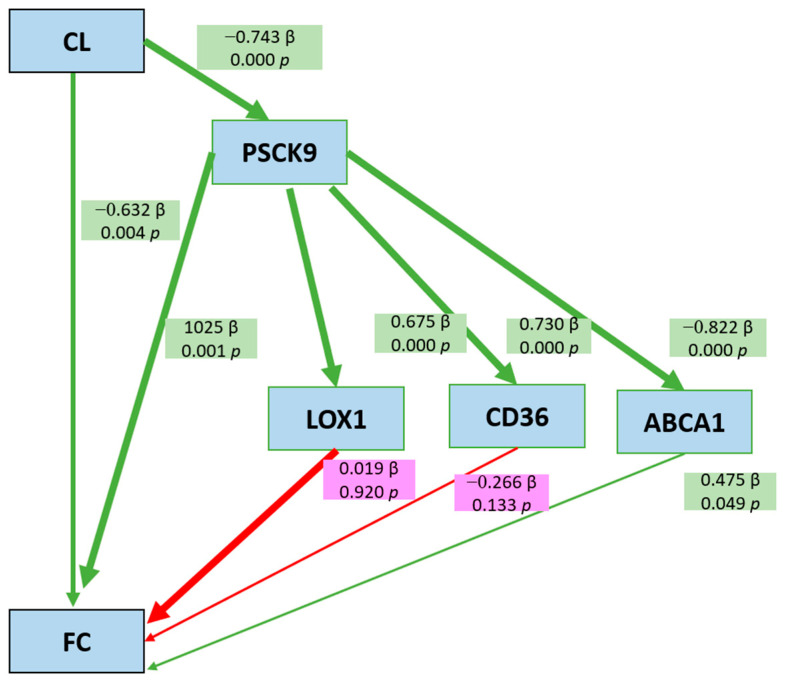
Path analysis of *Citrullus lanatus* (CL)’s influence on foam cell formation (FC). The figure illustrates the regression pathways between *Citrullus lanatus* (CL) extract and key atherogenic markers, namely PCSK9, ABCA1, LOX-1, CD36, and foam cell (FC) formation, in a rat model of atherosclerosis. Arrows indicate the direction of the relationships, with green arrows denoting statistically significant paths (*p* < 0.05) and red arrows representing non-significant paths (*p* > 0.05). Regression coefficients (β) and *p*-values are provided along each path. CL extract showed a significant negative effect on PCSK9 (β = −0.743, *p* = 0.000) and foam cell formation (β = −0.632, *p* = 0.004), and indirectly reduced FC formation through PCSK9 and ABCA1 (β = 0.290, *p* = 0.000). No significant indirect effects were observed through LOX-1 (β = −0.019, *p* = 0.221) or CD36 (β = −0.266, *p* = 0.133). These findings suggest that the PCSK9-ABCA1 pathway plays a dominant role in modulating foam cell formation, while LOX-1 and CD36 contribute minimally. The model explained 71.7% of the variance in FC formation (R^2^ = 0.717). Regression analysis was conducted using IBM Statistical Package for the Social Sciences (SPSS) Type 27 for Windows (IBM Corp., Armonk, NY, USA).

Our path analysis indicated that red watermelon extract influences the expression of PCSK9 and ABCA1, which are directly associated with foam cell formation. ABCA1 plays a critical role in reverse cholesterol transport by facilitating cholesterol efflux to apolipoprotein A-I (apoA-I), leading to the formation of HDL (Figure 4). This process is essential in preventing cholesterol buildup in the macrophages. It indicated that the ABCA1 pathway was the most dominant due to several factors. ABCA1 is a key protein in cholesterol transport that facilitates the efflux of cholesterol from cells to HDL particles, which is essential for reverse cholesterol transport (RCT). This pathway suggests that the experimental conditions promoted ABCA1 expression or activity, enhancing cholesterol removal from cells and preventing lipid accumulation in the macrophages [26,27]. External factors, such as antioxidants, nutraceuticals, or bioactive compounds, often increase the expression of ABCA1 through signaling pathways involving LXR or PPARγ. This pathway supports the role of ABCA1 in lipid metabolism [28]. LXR is a nuclear receptor that acts as a cholesterol sensor. When activated by oxysterols (cholesterol metabolites) or certain compounds such as antioxidants, LXR induces ABCA1 expression and mediates cholesterol efflux from cells to apolipoprotein A-I (apoA-I) to form nascent HDL. LXR activation reduces cholesterol buildup in the macrophages, thus reducing the risk of foam cell formation and atherosclerosis. Some studies suggest that although PCSK9 regulates cholesterol homeostasis, it is better known for its role in lowering LDL receptor (LDLR) expression, which impacts plasma cholesterol rather than directly impacting on cellular cholesterol effusions. In contrast, LXR/ABCA1 plays a more direct role in the regulation of intracellular free cholesterol [26,27,29]. Similarly, PPARγ also affects the expression of ABCA1. Polyunsaturated fatty acids or pharmacological agents such as PPAR agonists activate PPARγ, which increases the transcription of the LXR gene, thereby indirectly increasing the expression of ABCA1. This pathway integrates lipid metabolism and anti-inflammatory effects [30]. Compared with CD36 or LOX-1, the ABCA1 pathway is more specific in regulating cholesterol efflux to HDL. This makes this pathway more dominant in conditions where stimulation of anti-atherogenic activity is prioritized [31]. OxLDL is a pro-atherogenic factor that induces oxidative stress and inflammation in macrophage cells. These cells respond by upregulating ABCA1 activity to expel excess cholesterol to HDL particles. This aims to reduce the toxic effects of oxidized cholesterol. HDL, specifically apoA-I, directly interacts with ABCA1 to form nascent HDL through cholesterol efflux. If HDL concentrations are high, ABCA1 activity will be more stimulated than other receptors such as CD36 or LOX-1, which are not directly related to HDL [32]. These findings support the hypothesis in Zhang’s research (2011) that lycopene activates the PPARγ-LXRα-ABCA1 pathway, which contributes to the reduction in total cell cholesterol levels and has the potential to prevent atherosclerosis. Thus, the evidence strengthens the argument that ABCA1 plays a dominant role in the mechanism of action of lycopene through activation of the PPARγ-LXRα-ABCA1 transcription pathway [33]. Thus, the dominance of the ABCA1 pathway can occur due to its unique role in the prevention of lipid accumulation, its regulation by external agents, and its efficient response to metabolic stimulation.

These observations are consistent with previous findings suggesting that lycopene activates the PPARγ-LXRα-ABCA1 axis, significantly contributing to the reduction in total cholesterol and the prevention of atherosclerosis. Thus, ABCA1’s prominent role is attributed to its capacity to prevent lipid accumulation, respond effectively to antioxidant stimulation, and maintain cholesterol homeostasis at the cellular level.

This research has several limitations that need to be considered. First, the analysis carried out is still qualitative, so the exact levels of lycopene, citrulline, and vitamin C cannot be known. Second, the relationship of these compounds to the NF-κB signaling pathway is still theoretical and has not been validated through direct biological tests. For this reason, the future direction of research can be focused on quantitative analysis of the main compounds, as well as testing their biological effects through in vitro and in vivo studies. Further research is also suggested to explore more specific molecular mechanisms, such as the effect of compounds on gene expression or inflammatory proteins mediated by the NF-κB pathway and the PPARγ-LXRα-ABCA1 transcription pathway.

## 5. Conclusions

Red watermelon has potential in atherosclerosis prevention by modulating the expression of PCSK9, LOX-1, CD36, ROS, TNFα, and ABCA1. The bioactive compounds in red watermelon can inhibit the absorption of OxLDL by macrophages, lower PCSK9 expression to prevent LDL receptor degradation, and increase cholesterol efflux through ABCA1 activity. These effects reduce the risk of atherosclerotic buildup formation. However, further studies are needed to confirm these mechanisms in humans and determine the effective dose for atherosclerosis prevention.

## Figures and Tables

**Table 1:** Comparative analysis of biochemical and histological parameters. Data are presented as the mean ± standard deviation (SD). K−: negative control; K+: positive control (atherosclerosis); P1, P2, P3: treatment groups with 500, 750, and 1000 mg/day/rat of red watermelon extract, respectively. P *: ANOVA significance value. Post hoc **: LSD test showing significant differences (*p* < 0.05) between P3 and other groups.

Parameter	K− Mean ± SD	K+ Mean ± SD	P1 Mean ± SD	P2 Mean ± SD	P3 Mean ± SD	P * (ANOVA)	Post Hoc ** (Significant Comparisons)
PCSK9	22.19 ± 3.14	30.85 ± 1.56	23.69 ± 3.17	19.64 ± 1.59	15.87 ± 1.93	0.000	P3 < K+, K−, P1, P2
LOX-1	24.97 ± 5.50	27.48 ± 1.54	24.61 ± 2.49	19.68 ± 4.17	14.77 ± 2.63	0.000	P3 < all groups
CD36	23.01 ± 4.31	29.07 ± 2.28	26.62 ± 2.82	19.51 ± 2.95	17.76 ± 4.18	0.000	P3 < K+, P1, P2
ABCA1	19.94 ± 1.57	14.32 ± 1.91	17.95 ± 0.80	21.37 ± 1.67	26.82 ± 1.49	0.000	P3 > all groups
Foam Cell	20.20 ± 4.22	24.33 ± 1.74	22.40 ± 3.93	20.03 ± 4.18	14.70 ± 3.36	0.002	P3 < all groups
Tunica Intima Thickness	40.90 ± 9.24	44.37 ± 7.41	34.99 ± 5.86	28.79 ± 4.98	26.99 ± 6.22	0.001	P3 < K+, K−, P1, P2
Cholesterol	51.74 ± 9.22	98.40 ± 9.84	65.89 ± 12.49	54.37 ± 8.19	53.68 ± 6.52	0.000	P3 < K+, P1
Triglyceride	49.32 ± 17.24	147.65 ± 13.38	73.04 ± 10.01	51.10 ± 9.90	39.59 ± 6.27	0.000	P3 < all groups
LDL	17.33 ± 3.77	28.24 ± 3.39	15.76 ± 3.03	14.57 ± 4.54	12.48 ± 1.89	0.000	P3 < K+, K−
HDL	44.00 ± 9.80	32.32 ± 3.76	51.25 ± 10.49	56.40 ± 10.69	69.27 ± 12.67	0.000	P3 > all groups
ROS	194.50 ± 25.92	242.95 ± 72.78	184.84 ± 34.88	167.66 ± 47.89	141.81 ± 36.14	0.014	P3 < all groups
TNF-α	20.92 ± 3.69	31.47 ± 3.39	21.69 ± 2.91	18.01 ± 4.39	14.50 ± 2.71	0.000	P3 < all groups

## Data Availability

The original contributions presented in this study are included in the article/Supplementary Materials. Further inquiries can be directed to the corresponding author(s).

## Supplementary Materials

The following supporting information can be downloaded at: https://www.mdpi.com/article/10.3390/cimb47060433/s1.
