# Supplementary material for: Effectiveness of Red Watermelon in Preventing Atherosclerosis Through the Role of Lipids, PCSK9, LOX-1, CD36, and ABCA1 in Wistar Rats
Source: Curr Issues Mol Biol. 2025 Jun 8;47(6):433. doi: 10.3390/cimb47060433 (PMC12191513; doi:10.3390/cimb47060433)
Supplement: Supplementary file 1 [file cimb-47-00433-s001.zip › cimb-3619132-supplementary.pdf]

Supplementary file

This supplementary file contains the MS/MS fragmentation spectra for major bioactive compounds identified in Citrullus lanatus extract. The spectra include precursor and product ion transitions, retention times, and characteristic fragmentation patterns.

Supplementary Figure S1a. MS/MS Fragmentation Spectrum of Lycopene in Citrullus lanatus Extract.

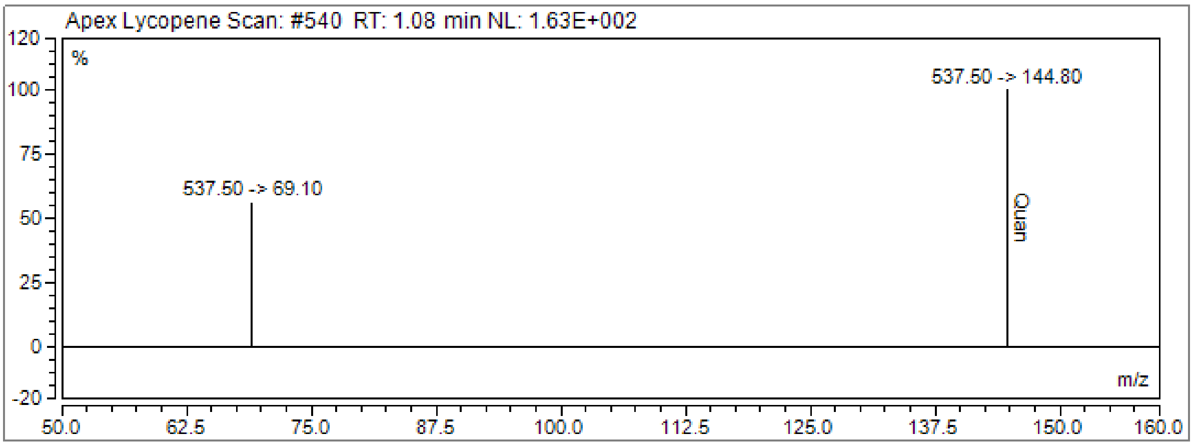

The spectrum shows the fragmentation pattern of Lycopene, with a precursor ion at m/z 537.50 and product ions at m/z 144.80 and 69.10. The analysis was performed by LC-MS/MS in positive ion mode. The x-axis represents the mass-to-charge ratio (m/z), and the y-axis indicates relative intensity (%). The retention time for Lycopene was 1.08 min, confirming its presence in the extract based on characteristic fragmentation patterns.

Supplementary Figure S1b. MS/MS Fragmentation Spectrum of Retinol (Vitamin A) in Citrullus lanatus Extract.

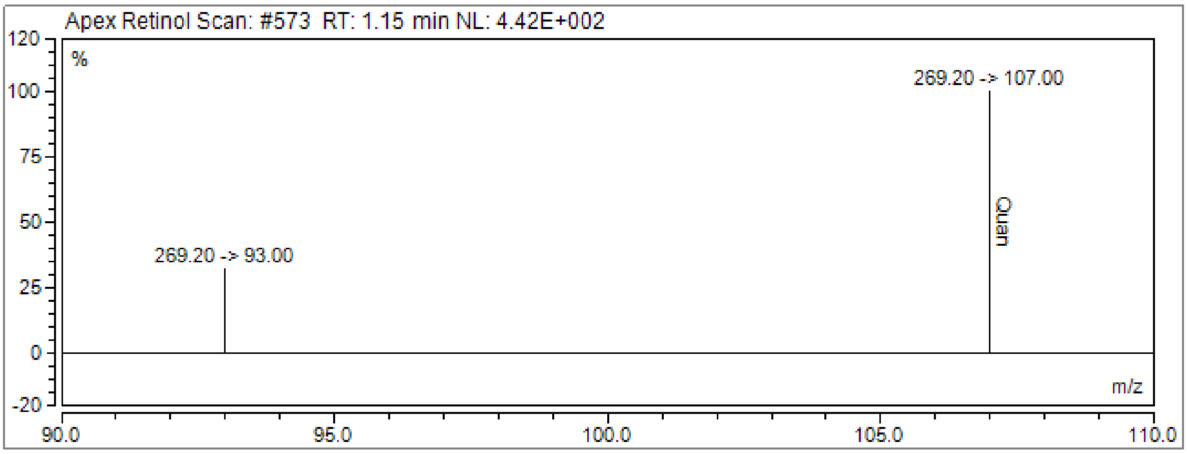

This spectrum displays the fragmentation pattern of Retinol, with a precursor ion at m/z 269.20 and product ions at m/z 107.00 and 93.00. The analysis was performed by LC-MS/MS in positive ion mode. The retention time for Retinol was 1.15 min. The spectrum confirms the identification of Retinol in the extract through characteristic fragment ions.

Supplementary Figure S1c. MS/MS Fragmentation Spectrum of Citrulline in Citrullus lanatus Extract.

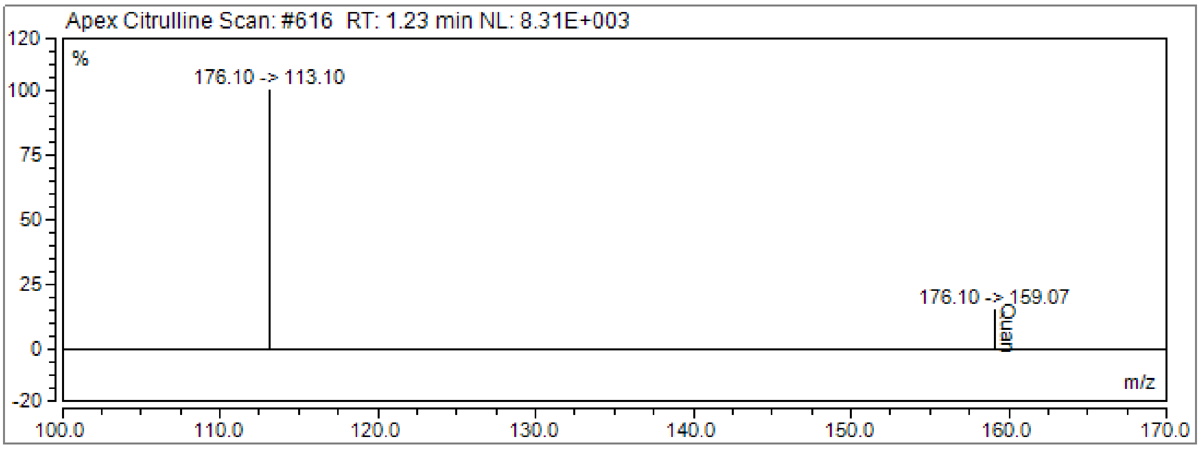

The MS/MS spectrum shows the precursor ion of Citrulline at m/z 176.10 and its product ions at m/z 113.10 and 159.07. The analysis was performed by LC-MS/MS in positive ion mode at a retention time of 1.23 min. The fragmentation pattern corresponds to the expected loss of ammonia and guanidino groups.

Supplementary Figure S1d. MS/MS Fragmentation Spectrum of Ascorbic Acid in Citrullus lanatus Extract.

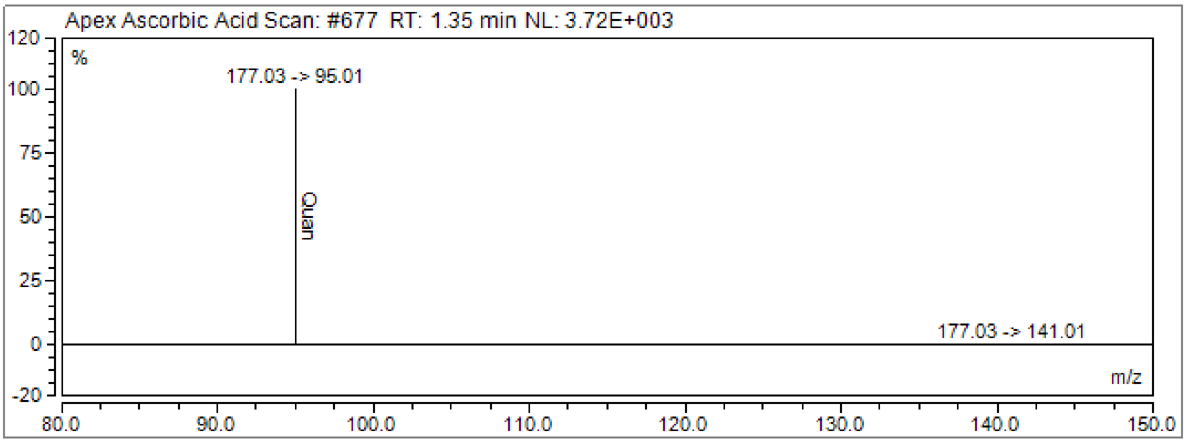

This figure shows the fragmentation spectrum of Ascorbic Acid, with a precursor ion at m/z 177.03 and product ions at m/z 95.01 and 141.01. The retention time was 1.35 min. The fragmentation confirms the presence of Ascorbic Acid based on characteristic dehydration and carboxyl group loss.

Supplementary Figure S1e. MS/MS Fragmentation Spectrum of α-Tocopherol in Citrullus lanatus Extract.

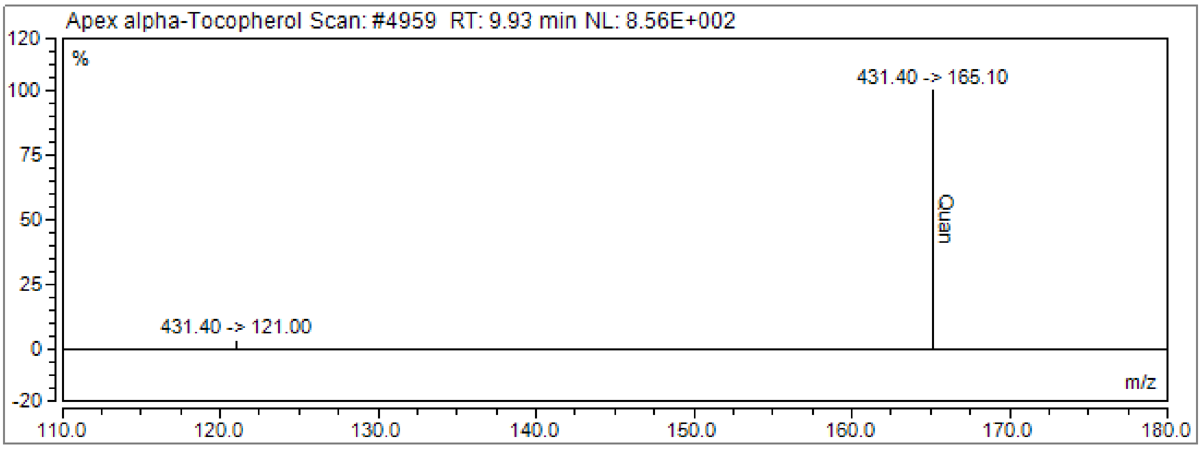

The spectrum shows the precursor ion of α-Tocopherol at m/z 431.40 and product ions at m/z 165.10 and 121.00. The analysis was performed by LC-MS/MS in positive ion mode at a retention time of 9.93 min. The fragmentation pattern matches known standards for α-Tocopherol, confirming its identification.
